# Prospective, Double‐Blind, Vehicle‐Controlled Assessment of Topical Bakuchiol on Photoaging in a Chinese Population

**DOI:** 10.1111/jocd.71000

**Published:** 2026-06-18

**Authors:** Zill‐e‐human Khan, Ratan K. Chaudhuri, Ajay Dulai, Anurag Tarmaster, Libing Pan, Raja K. Sivamani

**Affiliations:** ^1^ Integrative Research Institute Sacramento California USA; ^2^ Sytheon (Now Part of Hallstar) Parsippany New Jersey USA; ^3^ Integrative Skin Science and Research Sacramento California USA; ^4^ SGS‐CSTC Standards Technical Services (Shanghai) Co, LTD Shanghai China; ^5^ Pacific Skin Institute Sacramento California USA; ^6^ Department of Dermatology University of California – Davis Sacramento California USA; ^7^ College of Medicine California Northstate University Elk Grove California USA

**Keywords:** Bakuchiol, photoaging, pigmentation, poresAsian, wrinkles

## Abstract

**Background:**

Aging skin is a universal complaint among diverse patient populations. It encompasses physiologic changes to the skin, such as skin laxity, dyspigmentation, fine lines, and wrinkles. However, different populations may show different signs of aged skin. For example, Asian skin in particular exhibits stronger dyspigmentation in comparison to Caucasian skin. There has been a growing interest in using bakuchiol as an alternative to retinoids in treating the appearance of aging skin. This study explores how topical bakuchiol can improve the appearance of aging skin in a Chinese population.

**Aims:**

This study evaluates the effects of topical bakuchiol on markers of aging skin, such as wrinkle severity, dyspigmentation, radiance, and pore size.

**Patients/Methods:**

In this 84‐day study, 73 participants were randomized to receive either topical bakuchiol or a placebo. Participants were evaluated at five visits: Baseline, Day 28, Day 56, and Day 84. Facial photography and analysis were performed. Clinical grading assessed wrinkle severity, hyperpigmentation, skin radiance, and pore appearance. Tolerability was assessed.

**Results:**

There was a significant reduction in wrinkle severity, hyperpigmentation, pore size, and improved radiance in the Bakuchiol group compared to the vehicle group in a Chinese population of women and men at days 24, 52, and 84.

**Conclusion:**

The use of topical bakuchiol improved the appearance of wrinkle severity, hyperpigmentation, skin radiance, and pore size in a Chinese population.

## 1 Introduction

Skin aging can be attributed to intrinsic mechanisms, such as cellular senescence and DNA damage, and extrinsic mechanisms, such as ultraviolet (UV) radiation, pollution, and other extrinsic factors [1, 2]. Both routes lead to physiological changes to the skin, such as fine lines, wrinkles, dyspigmentation, loss of radiance, and skin laxity across diverse populations despite apparent differences in aging patterns [1, 2]. Compared to Caucasian women, Chinese women have a delay in wrinkle onset by about 10 years but exhibit more rapid aging between the ages of 40–50. They exhibit stronger dyspigmentation and a higher wrinkling rate over the age of 40 in comparison to Caucasian women [3, 4].

Retinoids are a class of medications that encompass natural and synthetic derivatives of vitamin A (retinol). In the past 20 years, retinoids have remained a gold standard in the industry for their ability to target skin concerns [5, 6, 7]. Topical retinoids can exhibit these effects at the molecular level by increasing collagen production, reducing collagen degradation, and stimulating cell turnover [7]. However, despite the wide range of benefits gained from regular retinoid usage, they come with significant side effects [8]. They can cause irritation, rash, and increased photosensitivity to the skin [8] and Asian populations are less tolerant to topical retinoids than Caucasian populations [9], Retinoids are also rather unstable and photosensitive in commercial products. One study demonstrated that some formulations reached up to 100% decline after one week of light exposure, and less than one‐third maintained > 80% of initial retinoid content at their shelf‐life after opening, regardless of the retinol content or product price [10]. Therefore, there is a push to seek alternatives to retinol, with no previous alternatives offering the same degree of benefit. Many patients are left dealing with the side effects or are forced to stop using the ingredient.

In recent years, skincare trends have increasingly favored botanical ingredients, driven by a growing demand for multifunctional products that target various skin concerns, including aging and even toning. Among these, bakuchiol has emerged as a promising topical anti‐aging ingredient, gaining attention for its wide range of skin benefits. It is a bioactive meroterpene that is extracted mainly from the *Psoralea corylifolia* (babchi) plant seeds and leaves [11]. It is further purified to yield a psoralen‐depleted bakuchiol with over 99% purity [12]. It has been used for centuries in Chinese and Ayurvedic medicine for its many health benefits, including its antimicrobial, antiviral, antitumorigenic, anti‐inflammatory, and antioxidative properties [13]. Interestingly, it was recently demonstrated that Bakuchiol has synergistic effects with ethyl (linoleate/oleate) on the endocannabinoid system (eCB) in the skin, which plays an active role in the regulation of skin inflammation [14, 15]. Additionally, the topical combination of these two ingredients has been shown to reduce cortisol levels. This suggests promising new avenues for modulating the eCB system in innovative skincare product development. Bakuchiol has been recognized as a functional retinol analog, offering similar benefits without the associated harsh side effects, and has consistently proven to be a strong contender for the title of the gold standard in anti‐aging skincare [12, 16]. It has demonstrated increased epidermal regeneration, skin hydration, radiance, combats hyperpigmentation, and reduces wrinkle severity [12, 17, 18, 19, 20, 21].

While previous studies have shown that bakuchiol can be helpful in the visible signs of photoaging [16, 22], full clinical studies have not been performed in populations with Asian skin types. In this study, we evaluated the effects of daily topical application of bakuchiol on the skin of Chinese women to better understand its effects on an Asian population. Specifically, this study investigates how the use of topical bakuchiol reduces characteristics of skin aging, including the appearance of wrinkle severity, dyspigmentation, radiance, and pore size.

## 2 Methods

### 2.1 Study Design

This study was a randomized, double‐blind, single‐center, placebo‐controlled clinical trial conducted over 84 days to evaluate the efficacy and tolerability of bakuchiol in improving facial signs of aging. Participants were randomized into two groups: The test product group and the placebo group. Both participants and investigators were blinded to treatment allocation.

### 2.2 Participants

A total of 73 Chinese male and female participants aged 32 to 60 years were enrolled, with 37 subjects in the test product group and 36 in the control group. Participants were selected according to the following inclusion and exclusion criteria. Subjects were healthy Asian males and females between 30 and 60 years of age. Subjects must have mild to moderate facial hyperpigmentation, and all skin types were included. Subjects were excluded if they had recently used vitamin C, vitamin A, or other medications that affect skin pigmentation and wrinkles. Subjects were excluded if they had any significant medical history or infections. The complete inclusion and exclusion criteria can be found in Supporting Information 1.

### 2.3 Intervention and Treatment Protocol

Participants underwent a 14‐day washout period before the study. During the study, they were instructed to apply the allocated product twice daily (morning and evening) using a pump dispenser delivering 0.3–0.4 g per application. A total of three containers (each lasting four weeks) were provided. Participants were required to document daily applications in a diary. The treatment group received 0.5% bakuchiol applied twice daily, while the control group received the same vehicle formulation without bakuchiol, also applied twice daily (Table 1).

**TABLE 1:** Ingredients in bakuchiol and control creams.

INCI name	Bakuchiol containing cream (% w/w)	Control cream (% w/w)
Water	X	X
Glycerin	X	X
Disodium EDTA	X	X
Butylene Glycol	X	X
Ethylhexylglycerin	X	X
Xanthan Gum	X	X
Caprylic/Capric Triglyceride	X	X
Phenethyl Benzoate	X	X
Glyceryl Stearate & PEG‐100 Stearate	X	X
Cetyl Alcohol	X	X
Hydroxyethyl Acrylate/Sodium Acryloyldimethyl Taurate Copolymer	X	X
Bakuchiol	X	

The bakuchiol used in this study was psoralen‐depleted and is commercially available under the trade name Sytenol A (INCI: Bakuchiol), with a purity of 99.5%. Sytheon prepared both the 0.5% bakuchiol‐containing lotion and the placebo formulation (without bakuchiol).

### 2.4 Assessment and Data Collection

Participants were evaluated at five visits: Baseline (D0), Day 28 (D28), Day 56 (D56), and Day 84 (D84). Assessments included:

### 2.5 Clinical Grading

Wrinkle severity, hyperpigmentation, skin radiance, and pore appearance were assessed using a modified Griffiths 10‐point scale (0 = best condition, 9 = worst condition).

### 2.6 Tolerability Assessment

Dermatological grading was used to assess erythema, edema, dryness/scaling, and peeling. Subjects reported burning, stinging, itching, tingling, and tightness. A four‐point Likert scale was used, which ranged from zero to three (none, mild, moderate, and severe).

### 2.7 Digital Imaging

- **VISIA‐CR Imaging:** Standardized facial photography under different lighting conditions (standard, cross‐polarized, parallel‐polarized) for pigmentation and pore analysis.
- **Primos CR Imaging:** 3D skin microtopography analysis for wrinkle count, depth, volume, and area on the forehead, crow's feet, and under‐eye areas.

### 2.8 Data Analysis

Statistical analyses were conducted using SPSS version 28.0. Changes from baseline were analyzed using Wilcoxon signed‐rank tests or paired *t*‐tests, depending on data distribution. Between‐group comparisons utilized Mann–Whitney *U* tests or independent *t*‐tests. Statistical significance was set at *p* < 0.05, and all values were reported with means, standard deviations, and percentage changes from baseline.

### 2.9 Adverse Events and Protocol Deviations

No adverse events were recorded. Protocol deviations, if any, were documented and reviewed by the investigator and sponsor.

## 3 Results

**FIGURE 1:**
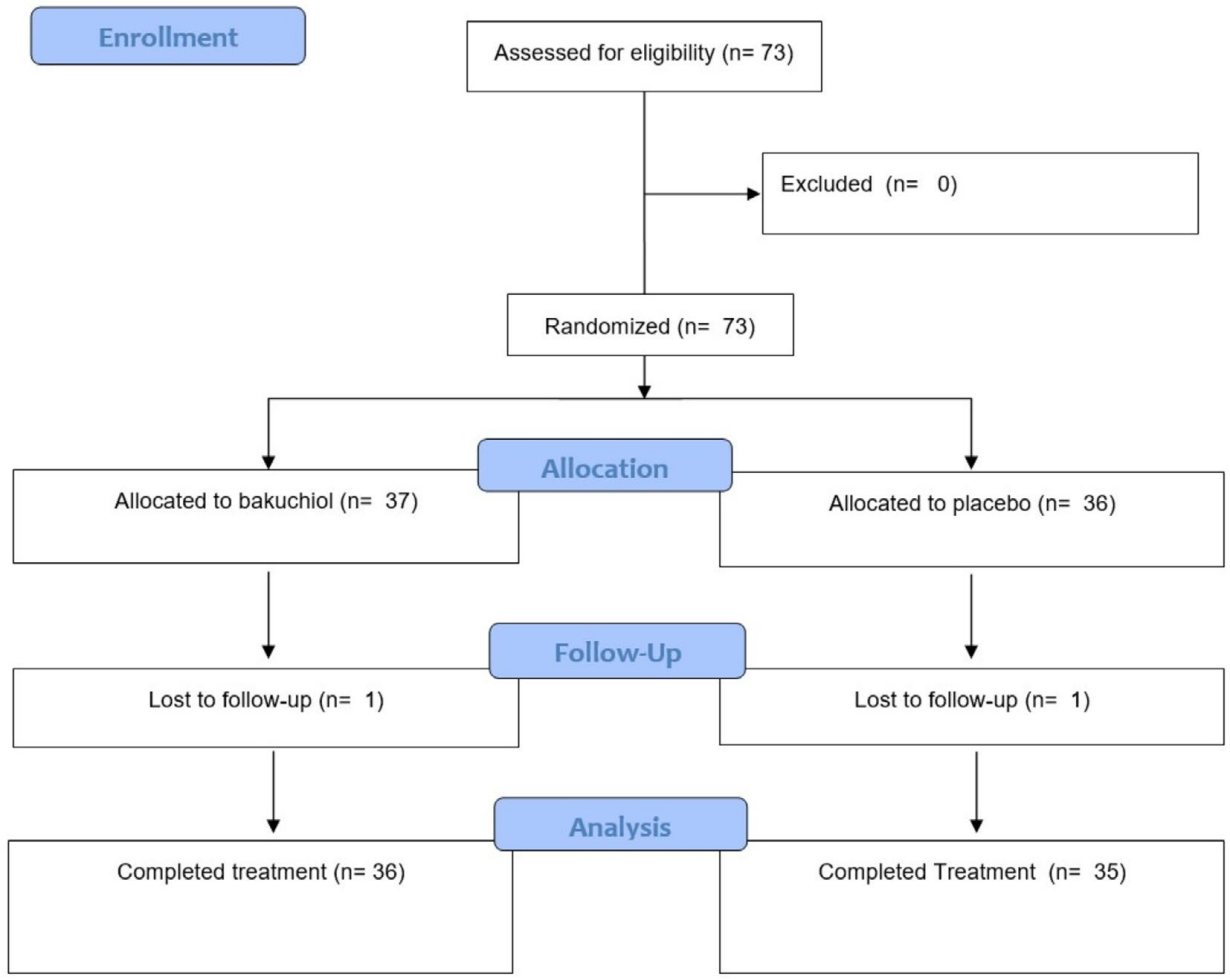
CONSORT (Consolidated Standards of Reporting Trials) flow diagram.

A total of 73 individuals were enrolled in this study (37 in the bakuchiol group and 36 in the control). A total of two subjects were lost to follow‐up, one from each treatment group (Figure 1). The average age of the experimental group was 50.33 (standard deviation (SD): 7.51) and 50.89 (SD: 7.21) for the control. Both groups were majority female in both the bakuchiol (34) and control (33) groups, with 2 males that were enrolled in each group.

### 3.1 Clinical Assessments

#### 3.1.1 Overall Wrinkles

**FIGURE 2:**
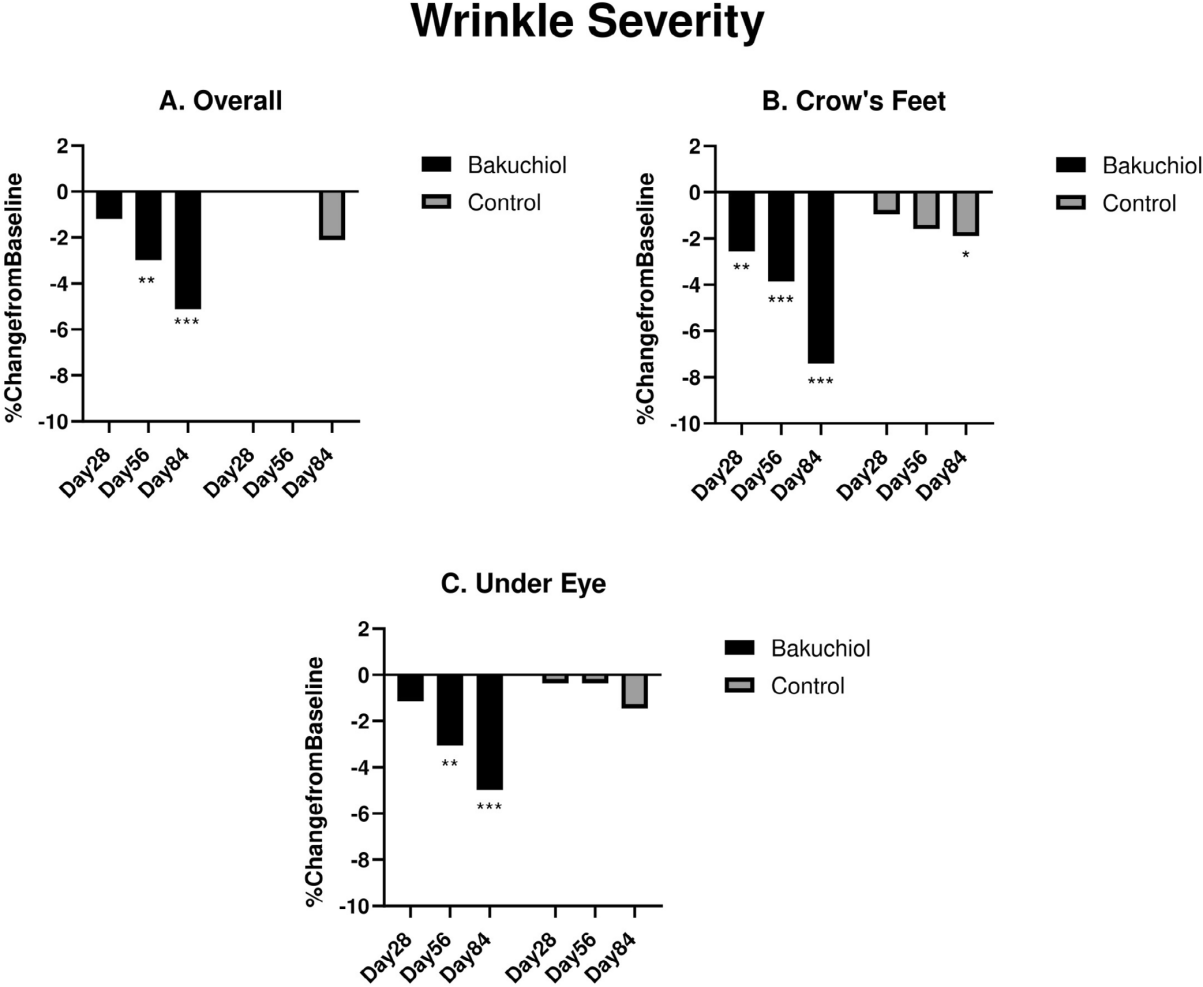
Wrinkle severity percent change in the active group and the placebo group at days 28, 56, and 84 compared to baseline. * = *p* < 0.05, ** = *p* < 0.01, *** = *p* < 0.001.

Relative global wrinkle reduction in the bakuchiol group was significant at day 56 (−3.0%, *p* = 0.002) and at day 84 (−5.2%, *p* < 0.001), but not at day 28 (Figure 2A). The control group demonstrated no significant change in wrinkles throughout the study. The bakuchiol group had a significant reduction in global wrinkles when compared to the control at both day 56 (*p* < 0.001) and day 84 (*p* = 0.023).

#### 3.1.2 Crow's Feet Wrinkles

The bakuchiol group demonstrated reductions in wrinkles on day 28 (−2.6%, *p* = 0.008), day 56 (−3.9%, *p* < 0.001), and day 84 (−7.4%, *p* < 0.001) (Figure 2B). The control group only noted a significant reduction at day 84 (−1.9%, *p* = 0.031). The bakuchiol had a significant reduction when compared to the control at day 84 (*p* < 0.001).

#### 3.1.3 Under Eye Wrinkles

The bakuchiol group demonstrated reductions starting from day 56: day 28 (−1.2%, *p* = 0.250), day 56 (−3.1%, *p* = 0.008), and day 84 (−5.0%, *p* < 0.001) (Figure 2C). The control group had no significant changes in wrinkles throughout the study. The bakuchiol group had a significant reduction relative to baseline at day 56 (*p* = 0.028) and day 84 (*p* = 0.040).

#### 3.1.4 Hyperpigmentation

**FIGURE 3:**
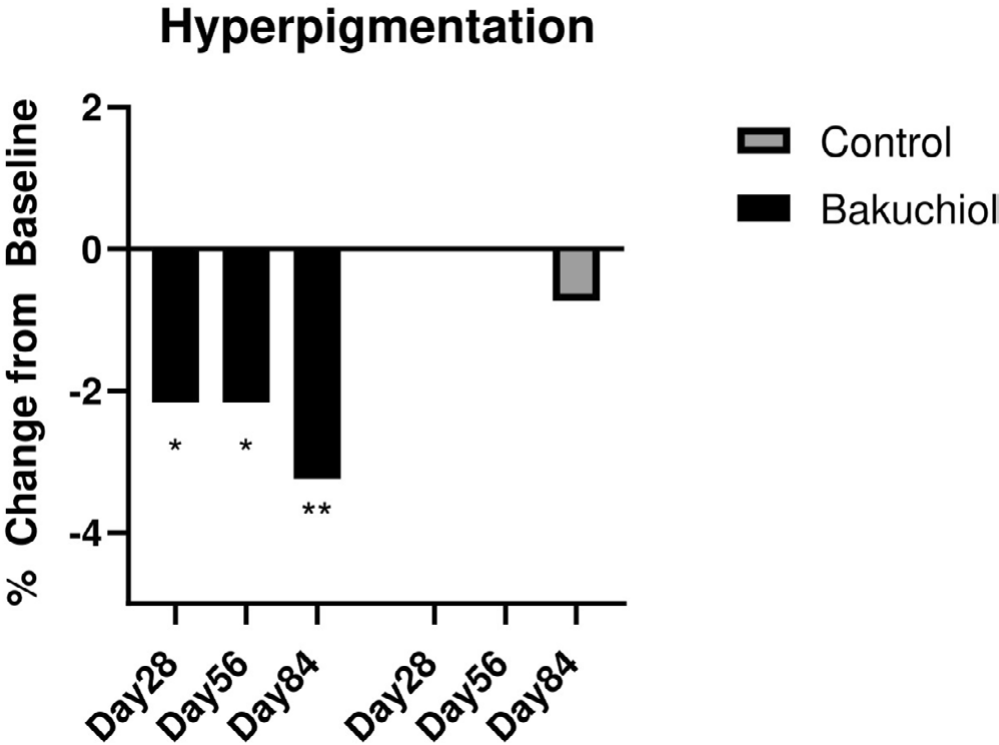
Hyperpigmentation severity percent change in the active group and the placebo group at days 28, 56, and 84 compared to baseline. * = *p* < 0.05, ** = *p* < 0.01.

Relative to baseline, reduction in hyperpigmentation was sustained during all visits in the bakuchiol group: day 28 (−2.2%, *p* = 0.031), day 56 (−2.16%, *p* = 0.031), and day 84 (−3.2%, *p* = 0.004) (Figure 3). The control group had no changes in hyperpigmentation at all three follow‐up visits (*p* > 0.05). The bakuchiol group had a significant reduction compared to control at day 28 (*p* = 0.025), day 56 (*p* = 0.025), and day 84 (*p* = 0.046).

#### 3.1.5 Skin Radiance

**FIGURE 4:**
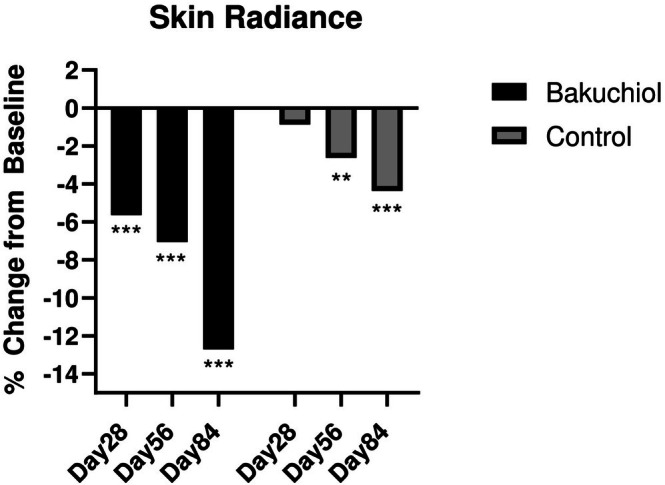
Skin radiance percent change in the active group and the placebo group at days 28, 56, and 84 compared to baseline. ** = *p* < 0.01, *** = *p* < 0.001.

The clinical assessment of skin radiance was reduced at all three follow‐up visits in the bakuchiol group: day 28 (−5.7%, *p* < 0.001), day 56 (−7.1%, *p* < 0.001), and day 84 (−12.7%, *p* < 0.001) (Figure 4). The control group only had a significant reduction relative to baseline in the latter two visits: day 28 (−8.4%, *p* = 0.250), day 56 (−2.6%, *p* = 0.004), and day 84 (−4.4%, *p* < 0.001); however, the bakuchiol group had a significant improvement compared to control at day 28 (*p* < 0.001), day 56 (p < 0.001), and day 84 (p < 0.001).

#### 3.1.6 Appearance of Pores

**FIGURE 5:**
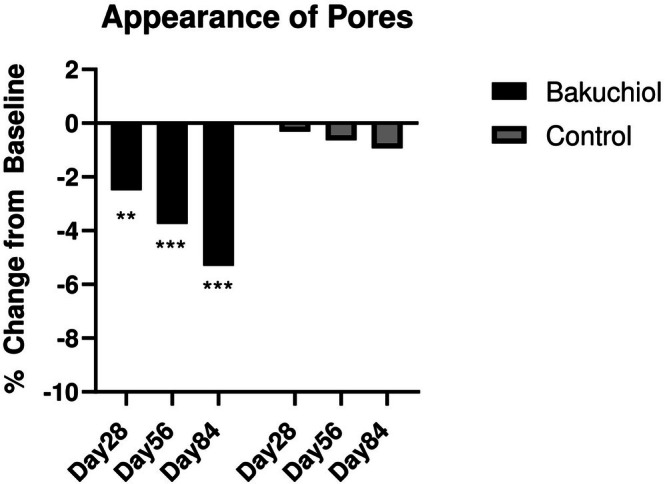
Pore size percent change in the active group and the placebo group at days 28, 56, and 84 compared to baseline. ** = *p* < 0.01, *** = *p* < 0.001.

The bakuchiol group had a significant reduction in the appearance of pores at all three follow‐up visits: day 28 (−2.5%, *p* = 0.008), day 56 (−3.8%, *p* < 0.001), and day 84 (−5.3%, *p* < 0.001) (Figure 5). The control group demonstrated no reduction at any point during the study (*p* > 0.05). The bakuchiol group significantly reduced the appearance of pores when compared to control: day 28 (*p* = 0.028), day 56 (*p* = 0.006), and day 84 (*p* < 0.001).

### 3.2 Tolerance Assessment

Results from the objective and subjective tolerance assessment rated on a Likert scale from zero to three in the bakuchiol group can be found in Table 2. Baseline values of subject tolerance assessment in tingling (0.06), stinging (0.06), dryness/scaling (0.36), itching (0.06), and burning (0.03) all reduced to 0 by day 84. Tightness had a 0.75 reduction by day 84 (−87.2%). Erythema, edema, and peeling were all assessed at baseline as 0 and remained 0 until day 84.

**TABLE 2:** Average score of objective and subjective tolerability assessment. scores were assessed from a likert scale of zero to three (none, mild, moderate, severe).

Parameter	Bakuchiol average Likert score			
	Day 0	Day 28	Day 56	Day 84
Tingling	0.06	0.03	0.00	0.00
Stinging	0.06	0.00	0.00	0.00
Dryness/Scaling	0.36	0.06	0.00	0.00
Erythema	0.00	0.00	0.00	0.00
Tightness	0.86	0.44	0.25	0.11
Itching	0.06	0.03	0.00	0.00
Peeling	0.00	0.00	0.00	0.00
Burning	0.03	0.00	0.00	0.00

### 3.3 Image Analysis

#### 3.3.1 Area of Pigmentation

The area of pigmentation improved in both groups, as assessed through image analysis. The bakuchiol group had a significant reduction at all three follow‐up visits: day 28 (−30.8%, *p* < 0.001), day 56 (−46.1%, *p* < 0.001), and day 84 (−60.0%, *p* < 0.001), and 100% of participants had some improvement. The control group similarly improved to a lesser extent but only at day 56 and day 84: day 28 (−4.9%, *p* = 0.152), day 56 (−18.4%, *p* < 0.001), and day 84 (−32.2%, *p* < 0.001). The experimental group had a significant improvement in pigmentation area when compared to the control at all visits: day 28 (*p* < 0.001), day 56 (*p* < 0.001), and day 84 (*p* < 0.001).

#### 3.3.2 Area of Pores

All follow‐up visits demonstrated a significant reduction in the area of pores in the bakuchiol group: day 28 (−4.8%, *p* < 0.001), day 56 (−9.6%, *p* < 0.001), and day 84 (−14.7%, *p* < 0.001). The control group demonstrated similar, but reduced improvement: day 28 (−2.8%, *p* = 0.008), day 56 (−5.3%, *p* < 0.001), and day 84 (−6.0%, *p* < 0.001). The bakuchiol group had significant reductions compared to control at day 56 (*p* = 0.024) and day 84 (*p* < 0.001), but not at day 28 (*p* = 0.262).

## 4 Discussion

In this 84‐day, prospective, randomized controlled trial of bakuchiol led to significant reductions in wrinkle severity, hyperpigmentation, pore size, and improved radiance were observed in a Chinese population of women and men at days 24, 52, and 84. Furthermore, this study supports the findings of previous studies that demonstrated bakuchiol as a safe and effective ingredient to improve signs of skin aging, such as wrinkles and hyperpigmentation [16, 22]. The result of this study expands the use of bakuchiol to reduce the appearance of wrinkles and hyperpigmentation in Asian skin.

The mechanism by which bakuchiol improves wrinkles and skin laxity can be attributed to its antioxidative, anti‐inflammatory, and collagen‐promoting properties, which allow for a multidimensional approach [12, 17]. Degradation of the extracellular matrix (ECM) of the dermis is critical in both intrinsic and extrinsic skin aging. UV exposure, inflammation, and matrix metalloproteases (MMPs) degrade collagen and ultimately contribute to the formation of wrinkles [2]. UV exposure accelerates the degradation of the ECM by forming reactive oxygen species; on the other hand, bakuchiol also exhibits antioxidant effects by multiple radical and non‐radical mediators of oxidative stress, such as prevention of mitochondrial lipid peroxidation, and activation of nuclear factor erythroid 2‐related factor 2 (Nrf2) [23, 24, 25, 26, 27]. Interestingly, one study demonstrated significance in bakuchiol's antioxidative and anti‐inflammatory [17]. Bakuchiol significantly reduced prostaglandin E2 (PGE2), macrophage migration inhibitory factor (MIF), increased FGF79 (fibroblast growth factor 7), and had significant wound healing properties through upregulation of fibronectin (FN) [17]. Additionally, a study from 2023 showed that bakuchiol + ethyl linoleate/oleate (BAK + ELN) worked synergistically to exert anti‐inflammatory effects in Tumor Necrosis Factor (TNF)‐stimulated human keratinocytes. Compared to cannabidiol (CBD), BAK + ELN was significantly more effective in inhibiting fatty acid‐binding protein 5 (FABP5) and reducing fatty acid amide hydrolase (FAAH). FABP5 and FAAH break down key endocannabinoids in the skin, and increased activation has been previously implicated in skin disease [28]. BAK + ELN further reduced inflammation through inhibition of the Cyclooxygenase (COX)‐2 pathway, decrease in type I interferon pathway genes, and reversal of the TNF‐induced shifts in gene expression [14]. Similarly, one study demonstrated that both bakuchiol and retinol reduced signs of photoaging, including wrinkle severity and hyperpigmentation, with no significant difference between the two groups. Previous studies have also demonstrated that bakuchiol enhances collagen production through increased expression of collagen type I, III, and IV mRNA [12, 13, 17, 29]. It has also been shown to upregulate the expression of inhibitors of tissue inhibitor matrix metalloproteinases (TIMP) 1 & 2, and reduce the expression of matrix metalloproteinase‐1 (MMP‐1) mRNA, which is involved in the breakdown of collagen 1 in the skin [12, 13, 17, 29]. Taken together, bakuchiol has many mechanisms by which it may improve photoaging and further supports the clinical findings of this study.

Interestingly, aging skin in Chinese women exhibits more hyperpigmentation than wrinkles [3, 4]. In our study, bakuchiol significantly reduced hyperpigmentation on days 28, 56, and 84, with improvements that were consistently greater than those in the control group at all time points. Image analysis revealed a significant reduction in the bakuchiol group on days 28, 56, and 84, with 100% of participants showing improvement. The control group also showed significant improvement, though to a lesser extent, on days 56 and 84. The reason for the improvements in the control group is not clear, but it may reflect a true effect of some of the components in the control vehicle cream. However, the bakuchiol group consistently demonstrated significantly greater improvement at all follow‐up visits. Bakuchiol is known to inhibit tyrosinase, the rate‐limiting enzyme in melanin synthesis [19, 20]. Our findings indicate that bakuchiol is both fast‐acting and effective in reducing pigment severity as early as day 28, supporting its potential anti‐aging and even toning skincare.

The observed improvement in pore size may be attributed to the activity of bakuchiol, highlighting its effectiveness even in an Asian population. Enlarged facial pores can be associated with seborrhea, aging, and skin laxity, and are a common aesthetic concern with age [30]. Multiple studies have shown that although Asian populations tend to have smaller pore sizes compared to other groups, the subjective concern about pore enlargement with age is equally prevalent [31, 32]. However, pores may be more apparent in Asian populations due to increased pigmentation around the pores, which makes them appear larger [33]. Bakuchiol may help reduce the appearance of pore size by promoting cell turnover, regulating sebum production, diminishing pigment severity, and enhancing skin elasticity and firmness through increased collagen and extracellular matrix protein (ECM) synthesis [7, 12, 17, 18, 19, 20, 21, 34, 35].

Our findings of high tolerability are consistent with earlier studies evaluating the tolerability of topical bakuchiol [16]. Minimal irritation was observed on day 24, and on day 86, all participants unanimously agreed that the product was well tolerated. No erythema, edema, or peeling was noted. A previous study demonstrated that bakuchiol was safe and effective in subjects with atopic dermatitis, eczema, rosacea, and cosmetic intolerance syndrome, which further supports its safety profile [18].

This study has several limitations. The findings reported here are limited to a Chinese population. Other studies have demonstrated effects in non‐Asian subjects [16, 22]. The population was predominantly women, with only two men recruited in each group. The current study results are likely applicable to a middle‐aged female population and cannot be generalized to the male population. This study does not directly compare the effects of bakuchiol against retinol. However, the use of a vehicle‐controlled design allows the effects of bakuchiol to be isolated and clearly highlighted. Our findings suggest that a future head‐to‐head comparative study between bakuchiol and retinol to assess both efficacy and tolerability is warranted. Environmental or behavioral factors were not limited as part of the study and may have served as a confounder, although any effect would have affected both the control and bakuchiol groups. Finally, this study was cosmetic in nature. Accordingly, the study results are limited to cosmetic endpoints, and structural or functional changes were not directly measured in this study. However, our studies suggest that future mechanistic studies are warranted. Furthermore, previous comparative studies have shown that bakuchiol is similar to retinol for its improvement of photoaging parameters, and further comparative studies in an Asian subpopulation are warranted.

## 5 Conclusion

Overall, this clinical study demonstrates that bakuchiol may improve several clinical parameters of photoaging in an Asian (Chinese) population that include improvement of the appearance of wrinkles in both the crow's feet and undereye regions, the appearance of facial hyperpigmentation, skin radiance, and the appearance of pore size.

## Author Contributions

Conceptualization, R.K.C., R.K.S.; Data curation, L.P.; Formal analysis, L.P., R.K.C., Z.K., A.D.; Funding acquisition, R.K.C.; Investigation, R.K.C., R.S.; Methodology, L.P.; Project administration, R.K.C., L.P.; Resources, R.K.C.; Supervision, L.P.; Writing – original draft, Z.K., A.D., A.T.; Writing – review and editing, Z.K., R.K.C., A.D., R.K.S., A.T., L.P.

## Funding

Funding was provided by Sytheon (now Hallstar).

## Ethics Statement

Subject recruitments were conducted with the approval of the SGS Ethics Committee for Clinical Research (approval number HZCPCH24001823). All participants provided written informed consent before participating in the study. The study was conducted in accordance with the Declaration of Helsinki, ensuring the protection of participants' privacy and confidentiality.

## Conflicts of Interest

R.K.S. is a consultant for Arbonne, Trace Minerals, Codex Labs, Burt's Bees, Image Skincare, Novartis, Sanofi, Bristol Myers Squibb, Pfizer, Nutrafol, Lilly, Arcutis, Janssen, Phothera, Galderma, Incyte, AbbVie, Leo, UCB, Sun, and Regeneron Pharmaceuticals.

## Supporting information


**Supporting Information: 1** Complete inclusion and exclusion criteria.

## Data Availability

The data that support the findings of this study are available on request from the corresponding author. The data are not publicly available due to privacy or ethical restrictions.
