# Supplementary material for: Prospective, Double‐Blind, Vehicle‐Controlled Assessment of Topical Bakuchiol on Photoaging in a Chinese Population
Source: J Cosmet Dermatol. 2026 Jun 18;25(6):e71000. doi: 10.1111/jocd.71000 (PMC13277955; doi:10.1111/jocd.71000)
Supplement: Supplementary file 1 — Supporting Information: 1 Complete inclusion and exclusion criteria. [file JOCD-25-e71000-s001.docx]

**Complete inclusion and exclusion criteria**

**Inclusion Criteria**

- Asian female or male, 30 to 60 years of age.
- In good general health (physical, mental, and social well-being, not merely the absence of disease/infirmity), according to subject self-report.
- All skin types.
- Mild to moderate facial hyperpigmentation, with a visual score of 3–6 (including 3 and 6) (SGS Atlas).
- Mild to moderate facial crow’s feet wrinkles, with a visual score of 3–6 (including 3 and 6) (SGS Atlas).
- Mild to moderate facial fine line (global face), with a visual score of 3–6 (including 3 and 6) (SGS Atlas).
- No recent tan (natural or artificial) on the area to be tested.
- Area free from hair, tattoos, scars, or beauty spots.
- Willing to provide written informed consent and able to read, speak, write, and understand Mandarin.
- Willing to cooperate and participate by following study requirements for the duration of the study and to report any changes in health status, medications, adverse event symptoms, or reactions immediately.

**Exclusion Criteria**

- Vitamin C or its derivative treatment within 1 month prior to this visit.
- Vitamin A or its derivative treatment within 1 month prior to this visit.
- Retinoid-based oral treatment within 6 months before or local treatment within 3 months prior to this visit.
- Any subject with an autoimmune disease.
- Subjects with clinically significant skin disorders that could compromise evaluation of reactions or may be aggravated by the application of the investigational product(s).
- Subject presenting with traces of dermal irritation, scars, active lesions (inflammatory disease), hyperpigmentation, moles, tattoos, pigmented marks, excessive pilosity, or any other abnormality on the concerned area that may interfere with the clinical evaluation.
- Nursing, pregnant, or planning to become pregnant during the study according to subject self-report.
- History of cancer or other serious/progressive disease/personal history of skin cancer or family history of melanoma, that could have an impact on the evaluated area, per the discretion of the investigator.
- Having a health condition and/or pre-existing or dormant dermatologic disease on the face (e.g., psoriasis, eczema, seborrheic dermatitis, severe excoriations) that the investigator or designee deems inappropriate for participation or could interfere with the outcome of the study.
- Having a history of immunosuppression/immune deficiency disorders (including HIV infection, AIDS, multiple sclerosis, Crohn’s disease, rheumatoid arthritis), organ transplant (heart, kidney, etc.), or currently using oral or systemic immunosuppressive medications and biologics (e.g., azathioprine, belimumab, Cimzia®, Cosentyx®, cyclophosphamide, cyclosporine, Enbrel®, Humira®, Imuran®, Kineret®, mycophenolate mofetil, methotrexate, Orencia®, prednisone, Remicade®, Rituxan®, Siliq™, Simponi®, Stelara®, Taltz®) and/or undergoing radiation or chemotherapy as determined by study documentation.
- Currently using or having regularly used corticosteroids (systemic or topical, not nasal or ocular) within the past 4 weeks.
- Having a disease such as asthma, diabetes, epilepsy, hypertension, hyperthyroidism, or hypothyroidism that is not controlled by diet or medication. Individuals having multiple health conditions may be excluded from participation even if the conditions are controlled by diet, medication, etc.
- Having started a long-term medication within the last 2 months.
- Having any planned surgeries or invasive medical procedures during the study. Non-invasive medical procedures or surgeries will be reviewed for their impact on the study outcome and acceptability by the investigator or designee.
- Currently participating in any other clinical study at SGS-CSTC Standards Technical Services Co., Ltd. Hangzhou Branch or at another research facility or doctor’s office.
- Having participated in any clinical study involving the test area within 2 weeks prior to inclusion into the study at SGS-CSTC Standards Technical Services Co., Ltd. Hangzhou Branch, or at another research facility or doctor’s office.
- Having started hormone replacement therapies (HRT) or hormones for birth control less than 3 months prior to study entry or who plan on starting, stopping, or changing doses of HRT or hormones for birth control during the study.
- Subject having applied anti-wrinkle, spot removal, and whitening products on the concerned areas during the 2 weeks preceding the starting day of the study.
